# Significant impact of cachexia index on the outcomes after hepatic resection for colorectal liver metastases

**DOI:** 10.1002/ags3.12578

**Published:** 2022-05-11

**Authors:** Yoshiaki Tanji, Kenei Furukawa, Koichiro Haruki, Tomohiko Taniai, Shinji Onda, Masashi Tsunematsu, Yoshihiro Shirai, Mitsuru Yanagaki, Yosuke Igarashi, Toru Ikegami

**Affiliations:** ^1^ Division of Hepatobiliary and Pancreas Surgery, Department of Surgery The Jikei University School of Medicine Tokyo Japan

**Keywords:** cachexia, colorectal liver metastases, liver resection

## Abstract

**Introduction:**

The purpose of this study was to investigate the relation between preoperative cachexia index (CXI) and long‐term outcomes in patients with colorectal liver metastases (CRLM) after hepatic resection.

**Method:**

In all,118 patients who underwent hepatic resection for CRLM were analyzed retrospectively. The relationship between CXI and the long‐term outcomes in patients after hepatic resection was investigated. CXI was calculated based on preoperative skeletal muscle index, serum albumin level, and neutrophil–lymphocyte ratio.

**Results:**

The multivariate analysis showed that extrahepatic lesion (hazard ratio [HR] 2.86, 95% confidence interval [CI] 1.48–5.53, *P* < .01) and high CXI (HR 0.44, 95% CI 0.20–0.98, *P* = .04) were independent and significant predictors of disease‐free survival. Moreover, extrahepatic lesion (HR 2.32, 95% CI 1.03–5.22, *P* = .04), high CXI (HR 0.17, 95% CI 0.05–0.57, *P* < .01), and curability R 1 or 2 (HR 3.29, 95% CI 1.23–8.78, *P* = .02) were independent and significant predictors of overall survival.

**Conclusion:**

CXI is a useful prognostic factor for disease‐free survival and overall survival after hepatic resection in CRLM patients.

## Graphical abstract

We investigate the relationship between preoperative CXI and long‐term outcome after hepatectomy in patients with colorectal liver metastases (CRLM), and this is the first report to show that CXI is a useful prognostic determinant after hepatectomy in patients with CRLM. #10;DFS and OS were predominantly prolonged in the group of patients with high CXI compared to the group of patients with low CXI.

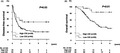

## 1 INTRODUCTION

Colorectal cancer is the most prevalent malignant tumor in Japan, with an increasing prevalence worldwide.^1^, ^2^ Overall, 20%–25% of these patients show liver metastases at the time of discovery, and 40%–50% develop colorectal liver metastases (CRLM) after primary lesion resection.^3^ Hepatic resection is the only treatment that offers the possibility of prolonging life, with a 5‐y survival rate of 33%–50%.^4^, ^5^ For improving prognosis, prognostic factors after hepatectomy for CRLM are important. Beppu et al reported prognostic prediction using six preoperative factors: synchronous metastases, primary lymph node positive, tumor number, tumor size, extrahepatic metastatic disease at hepatectomy, and preoperative carbohydrate antigen 19‐9 level.^6^ Recent reports suggest that nutritional factors, independent of tumor staging, such as the neutrophil–lymphocyte ratio (NLR), Glasgow Prognostic Score (GPS), prognostic nutrient index (PNI), and platelet–lymphocyte ratio (PLR) predict cancer‐specific survival in various cancers, including CRLM.^7^, ^8^ In addition, there are also reports on the relationship between sarcopenia and overall survival (OS) and disease‐free survival (DFS) for CRLM.^9^, ^10^

Cachexia is a complex metabolic disorder characterized by muscle loss with or without loss of fat mass associated with the underlying disorder and associated with more than 50% of cancer deaths and causes death in ~30%.^11^ Recently, the “cachexia index (CXI)” has been established, which consists of skeletal muscle index (SMI), serum albumin (Alb) level, and NLR, and may thus comprehensively reflect cachectic status.^12^, ^13^, ^14^, ^15^ However, the impact of CXI on the prognosis for digestive cancers has never been reported.

In this study we examined the possibility that CXI could be a better prognostic factor for CRLM, since sarcopenia and NLR have already been reported as prognostic factors for CRLM.

## 2 METHODS

### 2.1 Patients

The study period was from May 2007 to October 2017 and included 118 consecutive patients who underwent initial hepatic resection for CRLM at the Department of Surgery, Jikei University Hospital, Tokyo, Japan. Patient data were recorded prospectively and analyzed retrospectively. This study was approved by the Ethics Committee of the Jikei University School of Medicine (27‐177). The requirement for acquisition of informed consent from patients was waived because of the retrospective design of this study and anonymized data.

### 2.2 Treatment and patient management

All 118 patients without unresectable extrahepatic tumors underwent hepatectomy regardless of the size, number, or location of liver metastases, as long as curative resection left sufficient residual liver. The extent of hepatic resection was determined based on the retention rate of indocyanine green at 15 min (ICG_R15_).^16^ Patients with an estimated residual liver volume of <30% underwent percutaneous transhepatic portal vein embolization.

Contrast‐enhanced computed tomography (CT) and gadoxetic acid‐enhanced magnetic resonance imaging (MRI) were performed for tumor staging according to the criteria of the Japanese Society of Colon Cancer. Liver metastasis was classified as novel H1‐H3 (nH1‐nH3).^17^ The novel H classification is as follows: nH1 is isolated lesions of 5 cm or less in size; nH3 is five or more lesions of any size; and nH2 as anything in between nH1 and nH3.

Postoperative complications included surgical site infections and Grade ≥ III complications according to the Clavien–Dindo classification. Surgical site infection was defined as a condition where purulent discharge was observed with or without microbiological evidence in the incision or in an organ or space.

Recurrence of colorectal cancer after hepatic resection for patients with CRLM was defined by newly detected local, hepatic, pulmonary, or extrahepatic tumors by ultrasonography, CT, or MRI with or without an increase in serum carcinoembryonic antigen (CEA) or carbohydrate antigen 19‐9. For recurrent liver or lung metastasis, repeated hepatic or partial lung resection, respectively, or systemic chemotherapy was performed. For local recurrence, tumor resection, radiotherapy, or systemic chemotherapy was performed. For systemic chemotherapy, the patients received infusional 5‐fluorouracil/l‐leucovorin with oxaliplatin (FOLFOX) and/or infusional 5‐fluorouracil/l‐leucovorin with irinotecan (FOLFIRI).

### 2.3 Cachexia index

A hemogram and chemistry profile were conducted preoperatively. CXI was calculated based on preoperative SMI, Alb, and NLR. The formula for calculating CXI is SMI/(height (m) × height (m)) × Alb/NLR. The SMI was calculated by measuring the major and minor diameters of the iliopsoas muscle at the third lumbar vertebra using preoperative CT images. The SMI was calculated using the formula: iliopsoas major axis (mm) × iliopsoas minor axis (mm) × π/100. The CXI cutoff value was determined by receiver operating characteristic curves 3 yy after surgery for OS. CXI = 22.90 for men and CXI = 16.58 for women were set as cutoff values.

### 2.4 Analysis of risk factors for recurrence DFS and OS

We investigated the association between clinicopathologic variables and DFS or OS after initial liver resection by univariate and multivariate analyses. The variables include regional lymph node metastases of primary colorectal cancer, novel H classification (nH1‐nH3), tumor size, extrahepatic lesion, resectability (resectable, borderline resectable, or unresectable), neoadjuvant chemotherapy, serum CEA level, CXI, SMI, GPS,^18^ serum Alb level, serum C‐reactive protein (CRP) level, PNI, PLR, NLR, intraoperative blood loss, and curability (R0‐R2).

Continuous variables were classified into two groups for the log‐rank test and the Cox proportional hazard regression model based on novel H classification for tumor size, GPS for Alb and CRP, and optimal cutoff values determined by a receiver operating characteristic analysis for CEA, SMI, PNI, PLR, NLR, and intraoperative blood loss.

In addition, we investigated the relation between clinical variables and CXI by univariate analysis. The variables include age, gender, body mass index (BMI), timing of tumor, neoadjuvant chemotherapy, extrahepatic lesion, tumor number, tumor size, serum CEA level, serum Alb level, serum CRP level, GPS, PNI, NLR, PLR, operation time, intraoperative blood loss, postoperative complications, curability, and adjuvant chemotherapy.

### 2.5 Statistical analysis

The data were expressed as the median (interquartile range). The Mann–Whitney *U‐* and Chi‐squared tests were used to compare the continuous and dichotomous variables, respectively. Multivariate analysis of DFS and OS was performed using the Cox proportional regression model with a backward elimination stepwise approach. The survival curve was calculated using the Kaplan–Meier method with the log‐rank test. All *P*‐values were considered statistically significant if they had an associated probability of <.05.

## 3 RESULTS

### 3.1 Patient characteristics

Patients' characteristics are summarized in Table 1. The median age was 66 yy, with a range of 60–75 yy, and 81 (68.6%) study participants were male. The primary tumor site was right‐sided colon in 31 patients (26.3%) and left‐sided colon in 87 patients (73.7%). The resectability at the time of CRLM diagnosis was resectable in 104 patients (88.1%) and borderline or unresectable in 14 patients (11.9%). Neoadjuvant chemotherapy was performed on 41 patients (34.7%). CXI was low in 93 of 118 patients (78.8%). The median follow‐up durations for DFS and OS were 1.01 and 3.03 yy, respectively. The 3‐y DFS and OS rates after hepatic resection for CRLM were 19.5% and 50.8%, respectively.

**TABLE 1:** Patients' characteristics

Variables	Patients (n = 118)
Age, yeas	66 (60–75)
Gender	
Female	37 (31.4%)
Male	81 (68.6%)
Body mass index, kg/m^2^	22.5 (20.3–24.4)
Primary tumor location	
Ascending colon	19 (16.1%)
Transverse colon	12 (10.2%)
Descending colon	10 (8.5%)
Sigmoid colon	36 (30.5%)
Rectum	41 (34.7%)
T factor	
T1	2 (1.7%)
T2	5 (4.2%)
T3	72 (61.0%)
T4	39 (33.1%)
Lymph node metastases	
Yes	74 (62.7%)
No	44 (37.3%)
Timing of tumor	
Synchronous	74 (62.7%)
Metachronous	44 (37.3%)
Novel H classification	
nH1	44 (37.3%)
nH2	6 (5.1%)
nH3	68 (57.6%)
Tumor size, mm	49 (19–45)
Resectability	
Resectable	104 (88.1%)
Borderline resectable	12 (10.2%)
Unresectable	2 (1.7%)
Neoadjuvant chemotherapy	
Yes	41 (34.7%)
No	77 (65.3%)
Serum CEA, ng/mL	11.1 (4.7–38.7)
CXI	
High	25 (21.2%)
Low	93 (78.8%)
Serum CEA, ng/mL	11.1 (4.7–38.7)

Abbreviations: CEA, carcinoembryonic antigen; CXI, cachexia index.

### 3.2 Univariate and multivariate analyses of clinicopathological variables in relation to DFS after hepatic resection for CRLM

We investigated the relationship between the clinicopathological variables and DFS after hepatic resection in patients with CRLM (Table 2). Univariate analysis showed a poor DFS in patients with lymph node metastases (*P* = .03), nH2 or 3 (*P* = .02), extrahepatic lesion (*P* < .01), borderline resectable or unresectable (*P* = .03), and low CXI (*P* = .03). In multivariate analysis, extrahepatic lesion (HR 2.86, 95% CI 1.48–5.53, *P* < .01) and high CXI (HR 0.44, 95% CI 0.20–0.98, *P* = .04) were independent and significant predictors of DFS.

**TABLE 2:** Univariate and multivariate analyses of clinicopathological variables in relation to disease‐free survival after hepatic resection for colorectal liver metastases

Variables	N	DFS univariate analysis		DFS multivariate analysis	
		Hazard ratio (95% CI)	*P*‐value	Hazard ratio (95% CI)	*P*‐value
Lymph node metastases					
Yes	74	1.68	.03		NS
No	44	(1.04–2.69)			
Novel H classification					
nH2, nH3	74	1.73	.02		NS
nH1	44	(1.08–2.77)			
Tumor size, mm					
≥50	25	0.93	.79		NS
<50	93	(0.54–1.61)			
Extrahepatic lesion					
Yes	19	2.10	<.01	2.86	<.01
No	99	(1.21–3.64)		(1.48–5.53)	
Resectability					
Borderline or unresectable	14	1.96	.03		NS
Resectable	104	(1.05–3.65)			
Neoadjuvant chemotherapy					
Yes	41	1.37	.17		NS
No	77	(0.88–2.15)			
Serum CEA, ng/mL					
≥10.7	60	1.20	.43		NS
<10.7	58	(0.77–1.86)			
CXI					
High	25	0.51	.03	0.44	.04
Low	93	(0.28–0.94)		(0.20–0.98)	
SMI					
High	87	0.7–	.14		NS
Low	31	(0.43–1.13)			
GPS					
1 or 2	31	1.35	.22		NS
0	87	(0.83–2.20)			
Serum Alb					
≥3.5	97	0.92	.77		NS
<3.5	21	(0.52–1.63)			
CRP					
≥1	17	1.62	.10		NS
<1	101	(0.91–2.88)			
PNI					
≥46	58	0.81	.34		NS
<46	60	(0.52–1.25)			
PLR					
≥146.6	62	1.55	.05		NS
<146.6	56	(0.99–2.40)			
NLR					
≥2.15	71	1.23	.37		NS
<2.15	47	(0.78–1.94)			
Intraoperative blood loss, mL					
≥400	67	1.49	.08		NS
<400	51	(0.95–2.35)			
Curability					
R1 or 2	16	1.80	.05		NS
R0	102	(0.99–3.29)			

Abbreviations: Alb, albumin; CEA, carcinoembryonic antigen; CI, confidence interval; CRP, C‐reactive protein; CXI, cachexia index; DFS, disease‐free survival; GPS, Glasgow Prognostic Score; NLR, neutrophil‐lymphocyte ratio; PLR, platelet‐lymphocyte ratio; PNI, prognostic nutrition index; SMI, skeletal muscle mass index.

### 3.3 Univariate and multivariate analyses of clinicopathological variables in relation to OS after hepatic resection for CRLM

**FIGURE 1:**
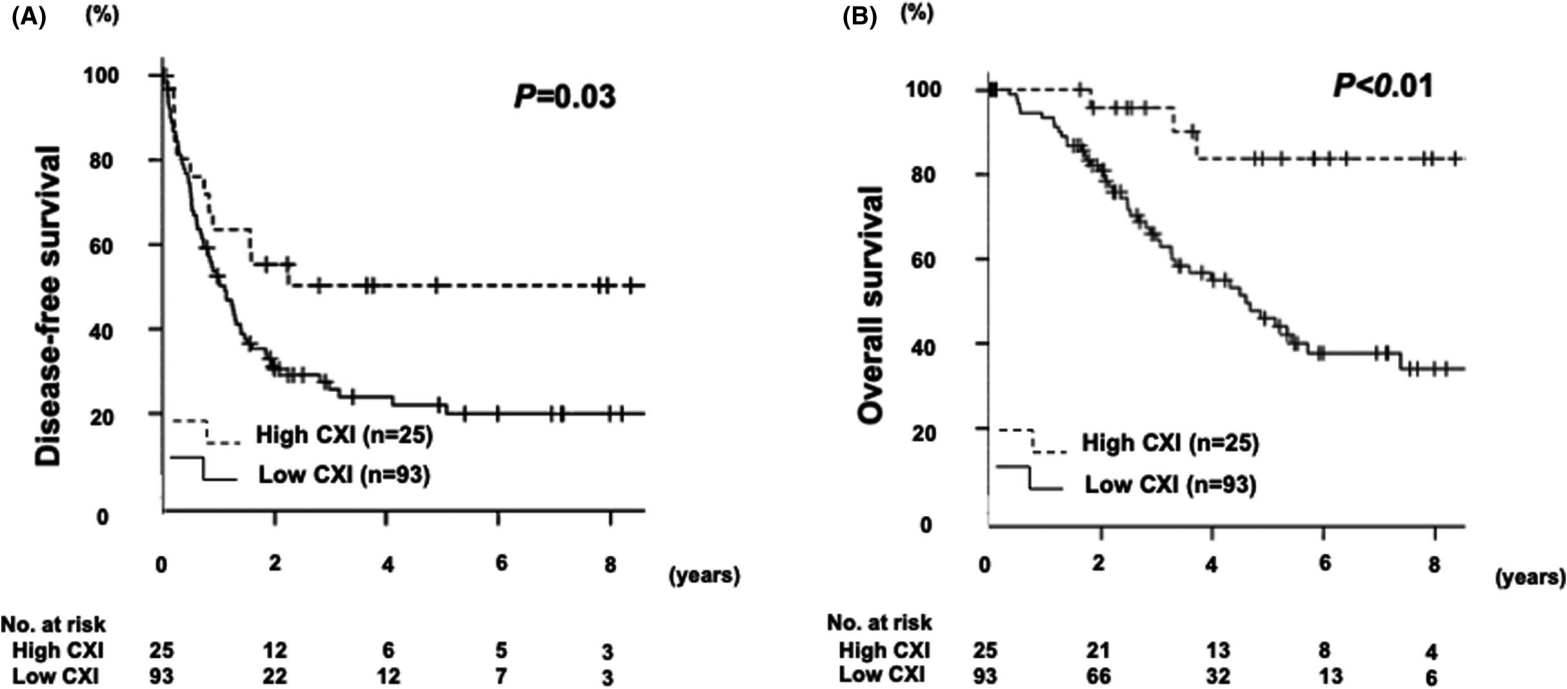
(A) Kaplan–Meier curve for disease‐free survival after hepatic resection for colorectal liver metastasis. (B) Kaplan–Meier curve for overall survival after hepatic resection for colorectal liver metastasis

We investigated the association between clinicopathological variables and OS after hepatic resection in patients with CRLM (Table 3). In univariate analysis, OS was poor in patients with extrahepatic lesion (*P* = .02), low CXI (*P* < .01), and R1 or 2 (*P* = .02). In multivariate analysis, extrahepatic lesion (HR 2.32, 95% CI 1.03–5.22, *P* = .04), high CXI (HR 0.17, 95% CI 0.05–0.57, *P* < .01), and R1 or 2 (HR 3.29, 95% CI 1.23–8.78, *P* = .02) were also independent and significant predictors of OS. Kaplan–Meier curve for DFS after hepatic resection for CRLM showed low CXI was significantly associated with worse DFS (*P* = .03; Figure 1). In addition, low CXI was also significantly associated with worse OS (*P* < .01; Figure 1).

**TABLE 3:** Univariate and multivariate analyses of clinicopathological variables in relation to overall survival after hepatic resection for colorectal liver metastases

Variables		OS univariate analysis		OS multivariate analysis	
	N	Hazard ratio (95% CI)	*P*‐value	Hazard ratio (95% CI)	*P*‐value
Lymph node metastases					
Yes	74	1.86	.06		NS
No	44	(0.98–3.50)			
Novel H classification					
nH2, nH3	74	1.46	.23		NS
nH1	44	(0.79–2.69)			
Tumor size, mm					
≥50	25	1.25	.52		NS
<50	93	(0.64–2.45)			
Extrahepatic lesion					
Yes	19	2.15	.02	2.32	.04
No	99	(1.12–4.14)		(1.03–5.22)	
Resectability					
Borderline or unresectable	14	1.76	.15		NS
Resectable	104	(0.82–3.76)			
Neoadjuvant chemotherapy					
Yes	41	1.64	.09		NS
No	77	(0.92–2.92)			
Serum CEA, ng/mL					
≥10.7	60	1.50	.17		NS
<10.7	58	(0.84–2.66)			
CXI					
High	25	0.22	<.01	0.17	<.01
Low	93	(0.08–0.63)		(0.05–0.57)	
SMI					
High	87	0.66	.19		NS
Low	31	(0.36–1.22)			
GPS					
1 or 2	31	1.73	.08		NS
0	87	(0.94–3.20)			
Serum Alb					
≥3.5	97	0.75	.43		NS
<3.5	21	(0.36–1.54)			
CRP					
≥1	17	1.72	.13		NS
<1	101	(0.86–3.46)			
PNI					
≥46	58	0.56	.05		NS
<46	60	(0.32–1.01)			
PLR					
≥146.6	62	1.53	.15		NS
<146.6	56	(0.86–2.71)			
NLR					
≥2.15	71	1.38	.31		NS
<2.15	47	(0.74–2.58)			
Intraoperative blood loss, mL					
≥400	67	1.34	.33		NS
<400	51	(0.75–2.43)			
Curability					
R1 or 2	16	2.53	.02	3.29	.02
R0	102	(1.18–5.45)		(1.23–8.78)	

Abbreviations: Alb, albumin; CEA, carcinoembryonic antigen; CI, confidence interval; CRP, C‐reactive protein; CXI, cachexia index; DFS, disease‐free survival; GPS, Glasgow Prognostic Score; NLR, neutrophil‐lymphocyte ratio; PLR, platelet‐lymphocyte ratio; PNI, prognostic nutrition index; SMI, skeletal muscle mass index.

### 3.4 Association between clinical variables and CXI

We investigated the association between clinical variables and CXI (Table 4). In univariate analysis, the low CXI group was significantly associated with older age (68 vs. 64 yy, *P* = .03), lower BMI (22 vs. 23 kg/m^2^, *P* = .02), lower Alb (3.8 vs. 4.0 g/dL, *P* < .01), higher CRP (0.08 vs. 0.21 mg/dL, *P* < .01), lower PNI (45 vs. 49, *P* < .01), higher NLR (2.6 vs. 1.4, *P* < .01), higher PLR (162 vs. 93.5, *P* < .01), and more postoperative complications (22 vs. 4%, *P* = .04). The timing of tumor, extrahepatic lesion, the tumor progression including tumor number and tumor size, CEA, and preoperative and postoperative chemotherapy were comparable between the two groups.

**TABLE 4:** Univariate analysis of clinical variables in relation to cachexia index

Variables	Cachexia index		*P*‐value
	High (n = 25)	Low (n = 93)	
Age, yeas	64 (56–70)	68 (61–75)	.03
Gender, female	7 (28%)	30 (32%)	.81
Body mass index, kg/m^2^	23 (22–26)	22 (20–24)	.02
Timing of tumor, synchronous	12 (48%)	62 (66.7%)	.11
Neoadjuvant chemotherapy, yes	10 (40%)	31 (33.3%)	.64
Extrahepatic lesion, yes	3 (12%)	16 (17.2%)	.76
Tumor number	2 (1–4)	2 (1–2)	.27
Tumor size, mm	21 (17–40)	27 (18–44)	.23
Serum CEA, ng/mL	9.7 (4.2–28)	12.4 (4.9–40)	.77
Serum Alb, g/dL	4.0 (3.8–4.3)	3.8 (3.5–4.1)	<.01
Serum CRP, mg/dL	0.08 (0.04–0.15)	0.21 (0.05–0.44)	<.01
GPS, 1 or 2	3 (12%)	28 (30.1%)	.08
PNI	49 (47–53)	45 (41–48)	<.01
NLR	1.4 (1.1–1.6)	2.6 (2.0–3.4)	<.01
PLR	93.5 (69.4–138)	162 (121–227)	<.01
Operation time, min	359 (307–436)	384 (275–496)	.61
Intraoperative blood loss, mL	490 (120–1050)	470 (190–1050)	.68
Postoperative complications, yes	1 (4%)	20 (22%)	.04
Curability, R1 or 2	3 (12%)	13 (14%)	1
Adjuvant chemotherapy, yes	16 (64%)	57 (61.3%)	1

Abbreviations: Alb, albumin; CEA, carcinoembryonic antigen; CRP, C‐reactive protein; GPS, Glasgow Prognostic Score; NLR, neutrophil‐lymphocyte ratio; PLR, platelet‐lymphocyte ratio; PNI, prognostic nutrition index.

## 4 DISCUSSION

In recent years, prognostic factors other than cancer staging, such as nutritional predictors and sarcopenia, have been studied. We demonstrated that CXI using NLR and SMI was a prognostic predictor of postoperative outcome in patients who underwent hepatic resection for CRLM. This is the first study to report that a low CXI is closely related to poor clinical outcomes in CRLM patients.

Cancer cachexia is a multifactorial syndrome characterized by a persistent loss of skeletal muscle mass (with or without loss of fat mass) that cannot be completely reversed with conventional nutritional support and leads to progressive functional impairment. In 2011, the European Palliative Care Research Collaborative proposed a new definition and classification of cancer cachexia, which is divided into three levels: pre‐cachexia, cachexia, and refractory cachexia.^19^ It has been proposed that various factors and inflammatory cytokines produced by the tumor and host affect different cells of the body, such as muscle cells, hepatocytes, and adipocytes, causing metabolic abnormalities that lead to cachexia.^20^ For instance, inflammatory cytokines produced by tumor cells, such as tumor necrosis factor‐α, interleukin (IL)‐6, and IL‐8, are thought to contribute to muscle wasting and atrophy by inducing oxidative stress in skeletal muscles and activating muscle degradation pathways.^21^, ^22^ Since cachexia has a complex pathophysiology, having a composite index to measure ongoing cachexia is essential rather than using a single index BMI.

The main clinical features of cancer cachexia are: poor nutritional status, systemic inflammation, and loss of muscle mass. Clinical indicators of these features include serum Alb, NLR, and SMI. Each of these has been independently associated with poor prognosis.^23^, ^24^, ^25^ Therefore, we decided that it would be beneficial to investigate whether CXI, along with the known factors, would serve as a better prognostic indicator for CRLM.

In this study the low CXI group had more elderly people with lower BMI, Alb, and PNI, and higher CRP, NLR, and PLR compared to the high CXI group. Hence, we considered that CXI reflects the status of PNI, NLR, and PLR and a comprehensive assessment of nutritional status. On the other hand, no significant difference in the number or size of tumors or the presence or absence of extrahepatic metastases co‐relating to the CXI values was observed. Also, there was no significant difference in surgical techniques such as operative time or intraoperative blood loss. This suggests that CXI reflects musculoskeletal status and nutritional status, not preoperative tumor progression or invasion, and does not contribute to the content of the surgical procedure. Although it has been reported that chemotherapy treatment is discontinued early and inpatient care is very common in the low CXI group due to treatment‐related toxicity,^26^ and preoperative chemotherapy induced skeletal muscle loss in patients with breast cancer,^27^ our study did not find a difference in the proportion of patients who received neoadjuvant chemotherapy or postoperative adjuvant chemotherapy between the low CXI and high CXI groups. In multivariate analysis, patients with extrahepatic lesions and those with low CXI showed a poor prognosis, in common with both DFS and OS. As a result, low CXI was demonstrated to be an independent and significant risk factor for poor DFS and OS after hepatic resection for CRLM. Moreover, patients with low CXI had more postoperative complications than those with high CXI. These results suggest that prevention or improvement of CXI decline may improve both short‐ and long‐term outcomes.

Several reports proposed that the CXI is a novel index for estimating cachexia that also correlates with prognosis for malignant tumors such as advanced non‐small cell lung cancer (NSCLC), non‐Hodgkin’s lymphoma, and diffuse large B‐cell lymphoma. In diffuse large B‐cell lymphoma, the low CXI group showed a predominant decrease in OS, and in patients with NSCLC and non‐Hodgkin’s lymphoma, the low CXI group showed a predominant decrease in both OS and DFS, which was similar to what was found for patients with CRLM in this study.^12^, ^13^, ^14^, ^15^ Therefore, CXI may be associated with treatment outcomes and prognosis in other cancers or diseases, and further research is needed.

Currently, a study is underway in Japan to improve cancer cachexia with the use of anamorelin, a ghrelin receptor agonist, that can be administered orally.^28^ In Japan, the use of anamorelin for cancer cachexia was approved on December 11, 2020 for four types of cancers: NSCLC, gastric cancer, pancreatic cancer, and colorectal cancer..^29^ Further clinical studies using anamorelin or novel medications are expected to promote research on improving CXI and the prognosis associated with it as well as to improve research on the clinical outcomes of CRLM.

The limitations of this study include its retrospective nature, the fact that it was conducted at a single university hospital, and the small number of patients. Another limitation is that the follow‐up period was relatively short. Therefore, a large‐scale prospective study is needed to validate the results.

Preoperative CXI is a reliable indicator to assess the DFS and OS of CRLM patients after hepatectomy. The use of this index for clinical decision making will allow a more accurate risk stratification system combined with tumor staging of CRLM. In conclusion, CXI seems to be a useful predictor of poor DFS and OS in patients with CRLM after hepatectomy.

## DISCLOSURE

Conflict of interest: The authors have no conflicts of interest or funding to declare.

Ethical approval: This study was approved by the Ethics Committee of the Jikei University School of Medicine (27‐177).

Informed consent: The requirement for acquisition of informed consent from patients was waived because of the retrospective design of this study and anonymized data.

Registry and the registration no. of the study/trial: N/A.

Animal studies: N/A.
